# Supplementary material for: Combination of Adult Mesenchymal Stem Cell Therapy and Immunomodulation with Dimethyl Fumarate Following Spinal Cord Ventral Root Repair
Source: Biology (Basel). 2024 Nov 20;13(11):953. doi: 10.3390/biology13110953 (PMC11591889; doi:10.3390/biology13110953)
Supplement: Supplementary file 1 [file biology-13-00953-s001.zip › biology-3216445-supplementary.pdf]

Supplementary Materials

Table S1. Details of antibodies used for flow cytometry.

Antibody	Fluorochrome	Supplier	Host	Catalog number	Concentration
**CD90**	PE-Cy7	BD	Mouse	561404	1:50
**CD54**	PerCP	BD	Mouse	554970	1:50
**CD45**	PE	BD	Mouse	559135	1:50
**RT1A**	PE	BD	Mouse	559993	1:50

Table S2. Antibodies used for the immunofluorescence and their respective supplier, host, catalog number and concentration.

Antibody	Supplier	Host	Catalog number	Concentration
GFAP	Abcam	rabbit	AB7260	1:750
Iba-1	Wako	rabbit	019-19741	1:750
Gad65	Abcam	mouse	AB26113	1:750
VGLUT-1	Synaptic Systems	rabbit	135303	1:1000
Synaptophysin	Synaptic Systems	rabbit	NBP2-25170	1:1000
Neurofilament	Millipore	rabbit	AB1989	1:1500
Alexa 594	Jackson	Anti-rabbit	711-585-152	1:400
Alexa 594	Jackson	Anti-mouse	715-585-150	1:400

Table S3. mRNA primers used for RT-qPCR followed by catalog number and target.

	Catalog number	Target
Vegfa	Rn01511602_m1	Vascular Endothelial Growth Factor
Bdnf	Rn02531967_s1	Brain-Derived Neurotrophic Factor
Hgf	Rn00566673_m1	Hepatocyte Growth Factor
Nos2	Rn00561646_m1	M1 Macrophage – pro-inflammatory
Arg1	Rn00691090_m1	M2 Macrophage – anti-inflammatory
Tnfa	Rn01525859_g1	Pro-inflammatory cytokine
Il6	Rn01410330_m1	Pro-inflammatory cytokine
Tgfb	Rn00572010_m1	Anti-inflammatory cytokine
Il4	Rn01456866_m1	Anti-inflammatory cytokine
Il13	Rn00587615_m1	Anti-inflammatory cytokine
Hprt1	Rn01527840_m1	Reference gene

Table S4. MSC cultures characterization. Percentage of staining obtained by flow cytometry for CD90, CD54, CD45 and RT1A for both MSCs lineages.

	CD90	CD54	CD45	RT1A
**AT-MSC**	99.98%	99.48%	0.53%	5.86%
**BM-MSC**	99.89%	92.01%	7.71%	2.43%

Table S5. Experiment I results: Neuronal survival analysis, GFAP, Iba-1 and synaptophysin immunolabeling (mean ipsi/contralateral ratios and corresponding SEM).

	DMEM	AT-MSCs	BM-MSCs
**Neuronal survival**	0.23 ± 0.04	0.56 ± 0.03	0.51 ± 0.04
**Synaptophysin**	0.51 ± 0.03	0.78 ± 0.03	0.62 ± 0.04

**GFAP**	2.75 ± 0.08	1.86 ± 0.14	2.06 ± 0.12
**Iba-1**	5.06 ± 0.43	2.35 ± 0.32	2.74 ± 0.29

Table S6. Percent ratio between the number of motoneurons present on the ipsilateral and contralateral side, as well as the ratio of integrated pixel density between the ipsilateral and contralateral sides for anti-GAD65, anti-VGLUT-1, anti-synaptophysin, anti-GFAP, and anti-IBA-1. Mean values per group ± standard error.

		+ AT-MSC	AT-MSC
**Neuronal survival**	0.44 ± 0.02	0.57 ± 0.02	0.63 ± 0.03
**GAD65**	0.81 ± 0.01	0.75 ± 0.01	0.76 ± 0.01
**VGLUT-1**	0.63 ± 0.03	0.80 ± 0.04	0.86 ± 0.01
**Synaptophysin**	0.78 ± 0.02	0.78 ± 0.02	0.91 ± 0.02
**GFAP**	2.23 ± 0.21	1.59 ± 0.13	1.14 ± 0.04
**Iba-1**	2.28 ± 0.06	1.59 ± 0.13	1.21 ± 0.06

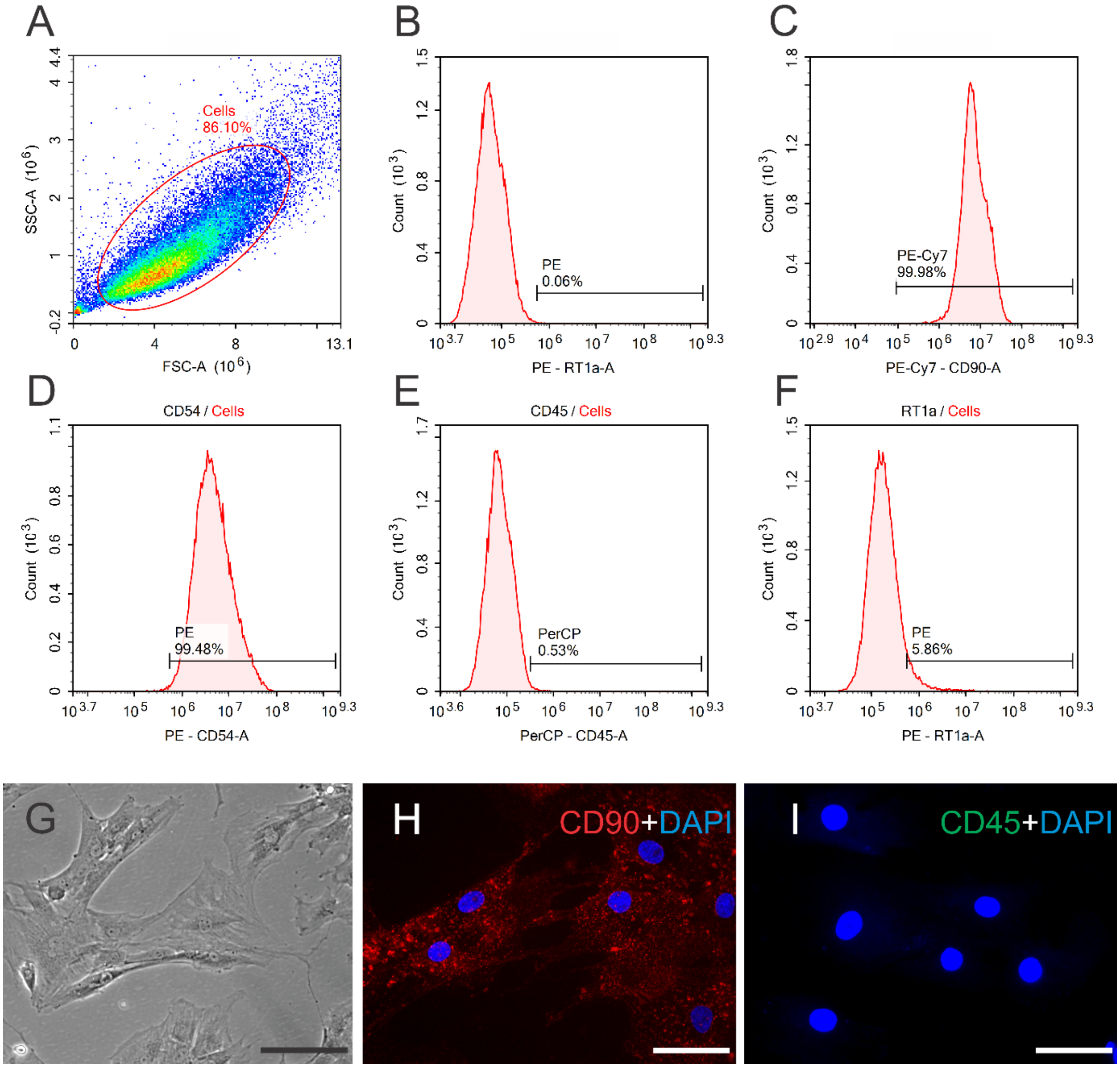

Figure S1. Characterization of AT-MSCs by flow cytometry. (A) Dot plot distribution of FSC-A vs. SSC-A used to gate the main cell population. (B) Histogram of the blank sample, followed by histograms showing positive expression of (C) CD90 and (D) CD54, and negative expression of CD45 (E) and RT1A (F). Phase contrast overview of AT-MSCs (G) (scale bar = 200 µm; 10x), with

immunostaining highlighting CD90 expression (H) and the absence of CD45 (I) (scale bar = 50 μm; 40x).

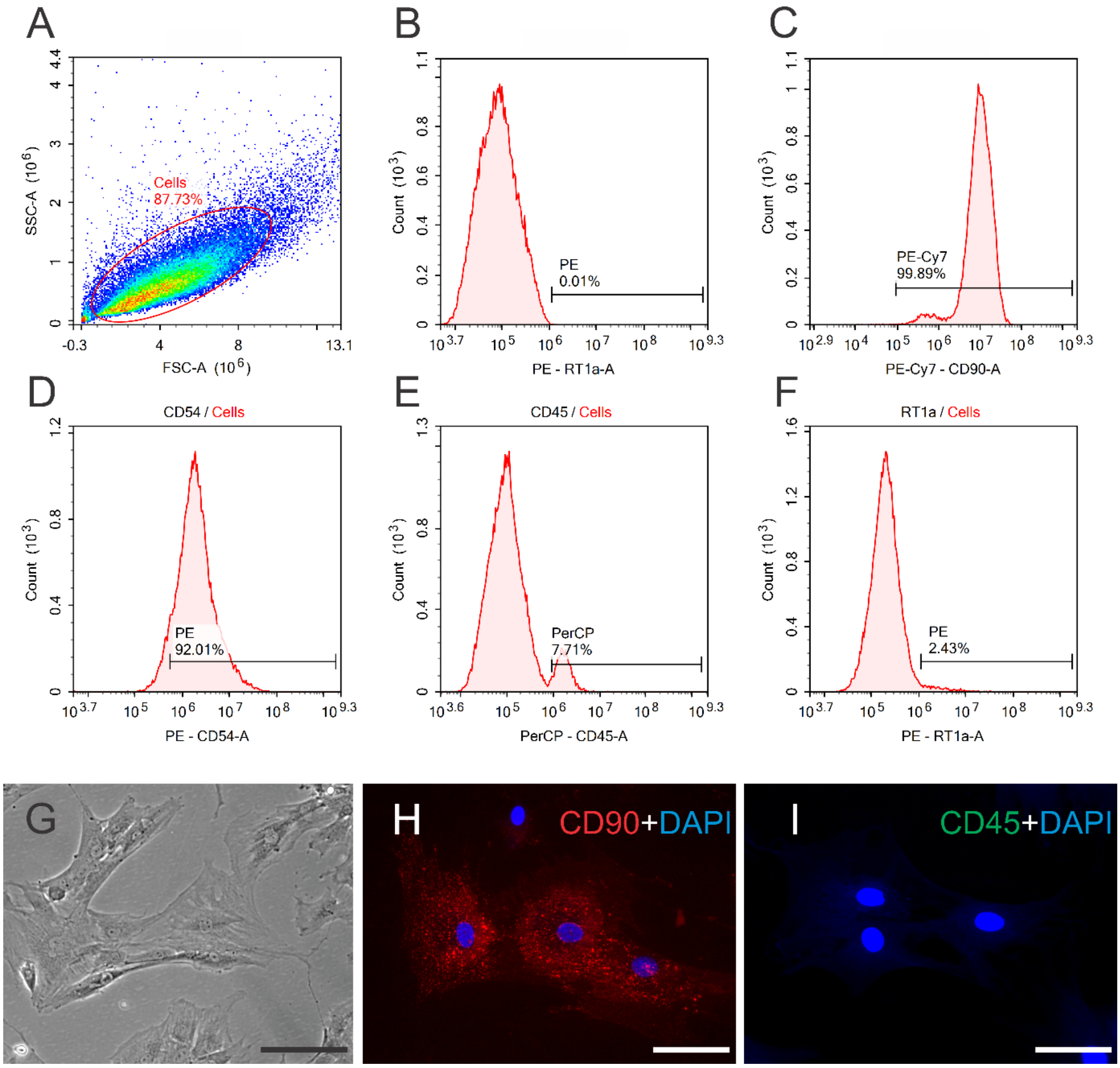

Figure S2. BM-MSC characterization by flow cytometry. (A) Dot-plot distribution of FSC-A vs. SSC-A used to gate the main cell population. (B) Histogram from the blank sample, followed by histograms depicting positive expression of (C) CD90 and (D) CD54, along with negative expression of (E) CD45 and (F) RT1A. Phase contrast overview of BM-MSC (G) (scale bar = 200 μm; 10x), with immunostaining highlighting CD90 expression (H) and the absence of CD45 (I) (scale bar = 50 μm; 40x).

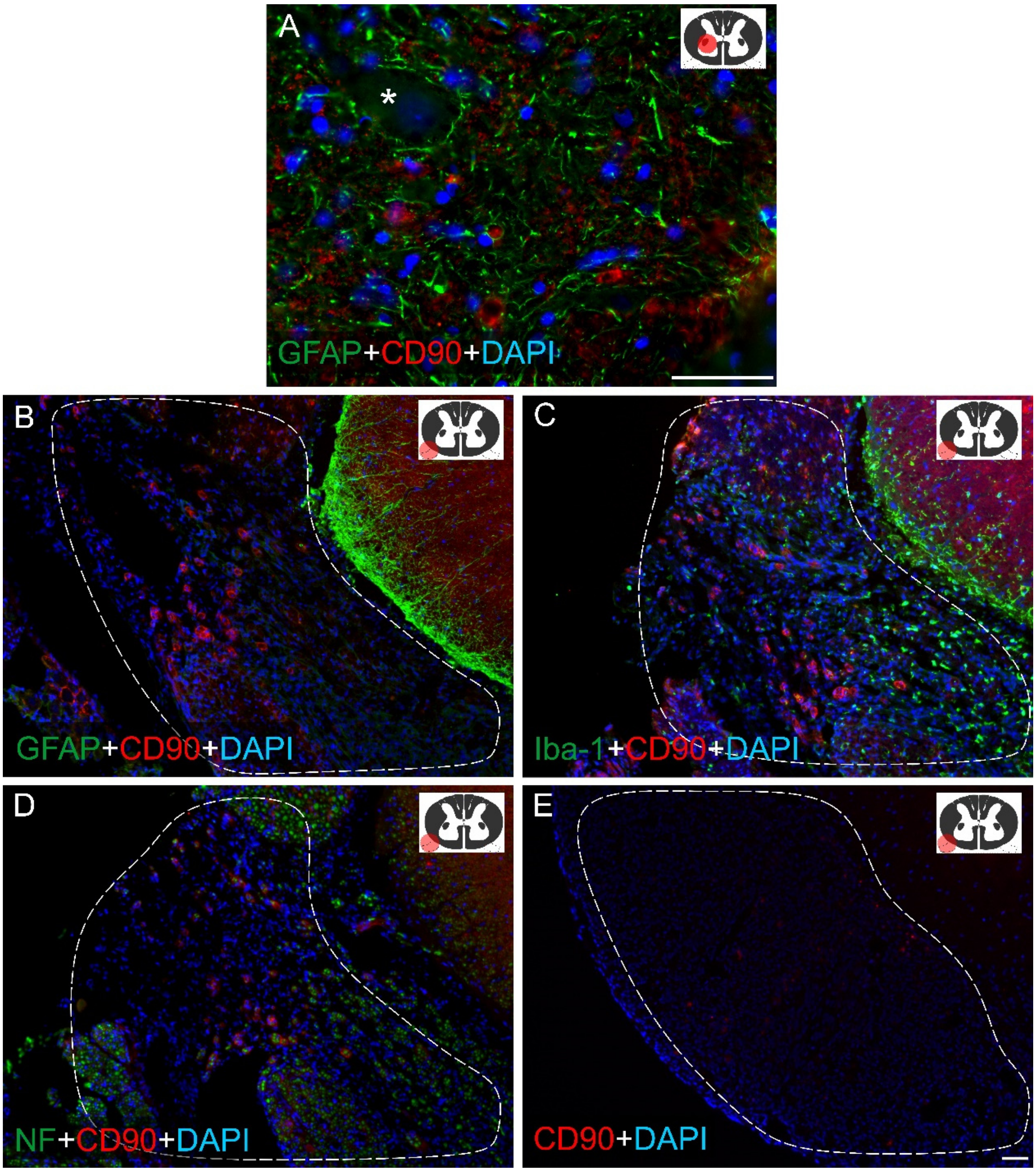

Figure S3. Localization of AT-MSCs within the re-implanted ventral roots (dashed lines) and lamina IX of Rexed, 4 weeks after coaptation with fibrin biopolymer. (A) CD90-positive AT-MSCs adjacent to spinal motoneurons (*) showing no co-localization with GFAP, an astrocyte marker. (B) CD90-labeled AT-MSCs within the reimplanted ventral root without co-localization with the astroglial marker GFAP. (C) Absence of co-localization of CD90-positive AT-MSCs with Iba-1, a microglial marker. (D) Neurofilament labeling of regenerating axons intermingled with undifferentiated CD90-positive AT-MSCs. (E) CD90 labeling in the control group that received root reimplantation but no cell treatment, indicating the possibility of a small amount of endogenous mononuclear cell migration to the site of injury. Scale bar = 50 μm.

Disclaimer/Publisher's Note: The statements, opinions and data contained in all publications are solely those of the individual author(s) and contributor(s) and not of MDPI and/or the editor(s). MDPI and/or the editor(s) disclaim responsibility for any injury to people or property resulting from any ideas, methods, instructions or products referred to in the content.
